# Supplementary material for: Contrasting effects of ocean acidification on tropical fleshy and calcareous algae
Source: PeerJ. 2014 May 27;2:e411. doi: 10.7717/peerj.411 (PMC4045329; doi:10.7717/peerj.411)
Supplement: Table S1 — The taxonomy and functional groupings of the algae used in CO2 experiments from 2009–2012. Sample size (n) is the number of replicates within a treatment per experiment, where each aquarium contained one alga. [file peerj-02-411-s001.docx]

**Table S1.** **Taxonomy and functional groupings of specimens.**

| Species | Phylum | Class | Order | n | Year |
| --- | --- | --- | --- | --- | --- |
| **Fleshy macroalgae** |  |  |  |  |  |
| *A. spicifera* (Vahl) Børgesen | Rhodophyta | Florideophyceae | Ceramiales | 6 | 2010 |
|  |  |  |  |  |  |
| *A. amadelpha* (Montagne)  Gepp & Gepp | Chlorophyta | Ulvophyceae | Bryopsidales | 10 | 2012 |
|  |  |  |  |  |  |
| *C. serrulata* (Forsskål) Agardh | Chlorophyta | Ulvophyceae | Bryopsidales | 6 | 2010 |
|  |  |  |  | 4 | 2011 |
|  |  |  |  |  |  |
| *D. bartayresiana* Lamouroux | Ochrophyta | Phaeophyceae | Dictyotales | 5 | 2011 |
|  |  |  |  |  |  |
| *H. pannosa* Agardh | Rhodophyta | Florideophyceae | Gigartinales | 5 | 2011 |
|  |  |  |  |  |  |
| **Upright calcareous algae** |  |  |  |  |  |
| *D. marginata* (Ellis & Solander) Lamarck | Rhodophyta | Florideophyceae | Nemaliales | 5 | 2011 |
|  |  |  |  |  |  |
| *G. rugosa* (Ellis & Solander) Lamarck | Rhodophyta | Florideophyceae | Nemaliales | 6 | 2010 |
|  |  |  |  |  |  |
| *H. opuntia* (Linnaeus) Lamouroux | Chlorophyta | Ulvophyceae | Bryopsidales | 4 | 2009 |
|  |  |  |  | 5 | 2011 |
|  |  |  |  |  |  |
| *H. taenicola* Taylor | Chlorophyta | Ulvophyceae | Bryopsidales | 4 | 2009 |
|  |  |  |  | 6 | 2010 |
|  |  |  |  | 10 | 2012 |
|  |  |  |  |  |  |
| **Crustose coralline algae** |  |  |  |  |  |
| *Lithophyllum* sp. Philippi | Rhodophyta | Florideophyceae | Corallinales | 4 | 2009 |
|  |  |  |  | 5 | 2011 |
|  |  |  |  |  |  |
| *L. prototypum* (Foslie) Foslie | Rhodophyta | Florideophyceae | Corallinales | 4 | 2009 |
|  |  |  |  |  |  |
