# Supplementary material for: Contrasting effects of ocean acidification on tropical fleshy and calcareous algae
Source: PeerJ. 2014 May 27;2:e411. doi: 10.7717/peerj.411 (PMC4045329; doi:10.7717/peerj.411)
Supplement: Table S2 — The design of CO2 enrichment experiments conducted on Palmyra Atoll from 2009–2012. In each experiment, handheld meters were used to monitor conditions, water baths to regulate temperature, and shading to reduce photo-inhibition, but the instruments and approaches varied by experiment. PAR: photosynthetically active radiation (µM photons m−2 s−1). [file peerj-02-411-s002.docx]

**Table S2.** **Experimental design of CO_2_ enrichment experiments.**

| Dates | Duration | Handheld meter | Calibration buffers for pH | Water bath type | Shade method | PAR |
| --- | --- | --- | --- | --- | --- | --- |
| 9/20/2009 to 10/3/2009 | 14 days | YSI Quatro^*^ | NIST, 3 points | Recirculating | Incident light | 150 ± 30 |
| 6/26/2010 to 7/5/2010 | 9 days | YSI Quatro | NIST, 3 points | Recirculating | Incident light | 178 ± 51 |
| 9/16/2011 to 10/2/2011 | 17 days | Hach^§^ | TRIS, 1 point | Flow-through | Direct w/shade cloth (1 layer) | 868 ± 72 |
| 9/14/2012 to 9/29/2012 | 15 days | Hach | TRIS, 1 point | Flow-through | Direct w/shade cloth  (2 layers) | 572 ± 20 |

* The YSI Environmental Quatro measures temperature (± 0.15 °C accuracy), salinity (± 0.1 ppt), pH (± 0.2), and dissolved oxygen using a polarographic electrode (± 0.2 mg L^-1^)

§ The Hach handheld meter measures temperature (± 0.3 °C accuracy), pH (± 0.1), and dissolved oxygen using an optode (± 0.01 mg L^-1^)
