# Supplementary material for: Contrasting effects of ocean acidification on tropical fleshy and calcareous algae
Source: PeerJ. 2014 May 27;2:e411. doi: 10.7717/peerj.411 (PMC4045329; doi:10.7717/peerj.411)
Supplement: Table S3 — The mean (±SE) carbonate system parameters of CO2 enrichment experiments conducted on Palmyra Atoll from 2009–2012. Water samples were collected at the same time of day (in duplicate after 2009) from a subset of blank control (no biological material), ambient air, or high pCO2 aquariums every 2–3 days; all analyses were conducted at 20 °C. Means were calculated by averaging control and treatment water samples over the duration of the experiment (only control samples were collected in 2009). AT, total alkalinity, CT, total inorganic carbon, pHSW, pH seawater scale, pCO2, partial pressure of CO2, ΩAr, saturation state of aragonite, ΩCa, saturation state of calcite. [file peerj-02-411-s003.docx]

**Table S3.** **Carbonate chemistry parameters for experiments conducted on Palmyra Atoll from 2009-2012.**

| Treatment | Species | | n | Salinity | A_T_  (µM kg^-1^) | C_T_  (µM kg^-1^) | pH_SW_ | pCO_2_  (µatm) | | | HCO_3_^-^  (µM kg^-1^) | | CO_3_^2-^  (µM kg^-1^) | | Ω_Ca_ | Ω_Ar_ | |
| --- | --- | --- | --- | --- | --- | --- | --- | --- | --- | --- | --- | --- | --- | --- | --- | --- | --- |
| **2009 Experiments** | | |  |  |  |  |  |  | | |  | |  | |  |  | |
| Ambient air | Control | | 3 | 34.8  (0.1) | 2221  (10) | 1994  (4) | 7.99  (0.02) | 440  (17) | | | 1817  (4) | | 163  (5) | | 3.9  (0.1) | 2.5  (0.1) | |
| High pCO_2_ | Control | | 3 | 34.8  (0.1) | 2236  (5) | 2140  (9) | 7.67  (0.03) | 1014  (75) | | | 2019  (11) | | 88  (5) | | 2.1  (0.1) | 1.4  (0.1) | |
| **2010 Experiments** | | |  |  |  |  |  |  | | |  | |  | |  |  | |
| Ambient air | Control | | 2 | 34.9 (0.1) | 2013  (24) | 1876  (16) | 7.81  (0.02) | 647  (20) | | | 1752  (12) | | 103  (5) | | 2.5  (0.1) | 1.6  (0.1) | |
|  | *A. spicifera* | | 3 | 34.8 (0.01) | 2132  (32) | 1984  (29) | 7.82  (0.01) | 659  (17) | | | 1850  (27) | | 113  (4) | | 2.7  (0.1) | 1.8  (0.1) | |
|  | *C. serrulata* | | 3 | 34.7 (0.1) | 2205  (30) | 1951  (9) | 8.04  (0.05) | 380  (42) | | | 1758  (18) | | 180  (18) | | 4.3  (0.4) | 2.8  (0.3) | |
|  | *G. rugosa* | | 3 | 34.7 (0.01) | 2148  (39) | 1977  (54) | 7.88  (0.06) | 589  (86) | | | 1831  (61) | | 127  (13) | | 3.0  (0.3) | 2.0  (0.2) | |
|  | *H. taenicola* | | 3 | 34.8 (0.03) | 1563  (85) | 1507  (104) | 7.56  (0.25) | 1330  (815) | | | 1405  (102) | | 59  (22) | | 1.4  (0.5) | 0.9  (0.3) | |
| High pCO_2_ | Control | | 2 | 34.8 (0.1) | 2058  (3) | 1994  (11) | 7.59  (0.05) | 1158  (129) | | | 1889  (14) | | 67  (7) | | 1.6  (0.2) | 1.0  (0.1) | |
|  | *A. spicifera* | | 3 | 34.8 (0.01) | 2224  (20) | 2237  (28) | 7.34  (0.14) | 2530  (906) | | | 2109  (11) | | 47  (12) | | 1.1  (0.3) | 0.7  (0.2) | |
|  | *C. serrulata* | | 3 | 34.8 (0.02) | 2215  (33) | 2072  (42) | 7.78  (0.12) | 818  (222) | | | 1931  (62) | | 115  (30) | | 2.8  (0.7) | 1.8  (0.5) | |
|  | *G. rugosa* | | 3 | 34.8 (0.04) | 2514  (143) | 2485  (113) | 7.46  (0.17) | 2215  (899) | | | 2342  (109) | | 71  (22) | | 1.7  (0.5) | 1.1  (0.3) | |
|  | *H. taenicola* | | 3 | 34.8 (0.04) | 1759  (91) | 1733  (82) | 7.45  (0.04) | 1365  (72) | | | 1646  (78) | | 44  (6) | | 1.0  (0.1) | 0.7  (0.1) | |
| **2011 Experiments** | | |  |  |  |  |  |  | | |  | |  | |  |  | |
| Ambient air | Control | | 2 | 35.1 (0.1) | 2256  (6) | 1952  (2) | 8.12  (0.01) | 306  (4) | | | 1729  (1) | | 213  (3) | | 5.1  (0.1) | 3.3  (0.04) | |
|  | *C. serrulata* | | 4 | 35.2 (0.04) | 2128  (22) | 1899  (44) | 7.99  (0.08) | 443  (109) | | | 1723  (59) | | 161  (20) | | 3.9  (0.5) | 2.5  (0.3) | |
|  | *D. bartayresiana* | | 4 | 35.1 (0.05) | 2107  (38) | 1821  (51) | 8.12  (0.03) | 296  (33) | | | 1616  (57) | | 195  (9) | | 4.7  (0.2) | 3.0  (0.1) | |
|  | *H. pannosa* | | 3 | 35.2 (0.1) | 2178  (18) | 1677  (100) | 8.42  (0.15) | 157  (76) | | | 1320  (168) | | 342  (71) | | 8.2  (1.7) | 5.3  (1.1) | |
|  | *D. marginata* | | 4 | 35.1 (0.03) | 1974  (105) | 1593  (151) | 8.30  (0.10) | 186  (64) | | | 1341  (173) | | 246  (26) | | 5.9  (0.6) | 3.8  (0.4) | |
|  | *H. opuntia* | | 4 | 35.1 (0.1) | 595  (68) | 596  (53) | 7.22  (0.24) | 1116  (470) | | | 546  (51) | | 13  (7) | | 0.3  (0.2) | 0.2  (0.1) | |
|  | *Lithophyllum* sp. | | 4 | 35.0 (0.01) | 1995  (50) | 1798  (38) | 7.95  (0.02) | 433  (13) | | | 1647  (29) | | 137  (9) | | 3.3  (0.2) | 2.1  (0.1) | |
| High pCO_2_ | Control | | 2 | 34.8 (0.1) | 2255  (7) | 2103  (3) | 7.82  (0.01) | 700  (12) | | | 1961  (1) | | 119  (2) | | 2.9  (0.1) | 1.9  (0.04) | |
|  | *C. serrulata* | | 4 | 35.2 (0.1) | 2125  (31) | 2023  (53) | 7.66  (0.16) | 1197  (362) | | | 1890  (73) | | 94  (33) | | 2.2  (0.8) | 1.5  (0.5) | |
|  | *D. bartayresiana* | | 3 | 35.2 (0.02) | 2128  (34) | 1903  (40) | 7.99  (0.06) | 431  (62) | | | 1731  (50) | | 159  (18) | | 3.8  (0.4) | 2.5  (0.3) | |
|  | *H. pannosa* | | 4 | 35.1 (0.03) | 2159  (37) | 1731  (135) | 8.24  (0.29) | 520  (418) | | | 1420  (218) | | 294  (98) | | 7.0  (2.3) | 4.6  (1.5) | |
|  | *D. marginata* | | 4 | 35.1 (0.04) | 1735  (100) | 1385  (153) | 8.30  (0.14) | 181  (77) | | | 1165  (178) | | 214  (33) | | 5.1  (0.8) | 3.3  (0.5) | |
|  | *H. opuntia* | | 4 | 35.0 (0.03) | 818  (131) | 870  (151) | 7.02  (0.14) | 2178  (816) | | | 791  (132) | | 8  (2) | | 0.2  (0.05) | 0.1  (0.03) | |
|  | *Lithophyllum* sp. | | 4 | 35.2 (0.04) | 2104  (38) | 2021  (22) | 7.63  (0.08) | 1108  (228) | | | 1907  (19) | | 79  (12) | | 1.9  (0.3) | 1.2  (0.2) | |
| **2012 Experiments** | |  | |  |  |  |  | |  |  | |  | |  | | |  |
| Ambient air | Control | | 4 | 35.0 (0.1) | 2262  (10) | 1957  (1) | 8.12  (0.01) | 316  (11) | | | 1733  (5) | | 214  (6) | | 5.1  (0.2) | 3.3  (0.1) | |
|  | *A. amadelpha* | | 2 | 35.2 (0.2) | 2124  (58) | 1922  (19) | 7.94  (0.08) | 486  (90) | | | 1762  (4) | | 144  (26) | | 3.4  (0.6) | 2.2  (0.4) | |
|  | *H. taenicola* | | 2 | 35.0 (0.04) | 1889  (139) | 1665  (36) | 8.00  (0.21) | 400  (183) | | | 1499  (24) | | 152  (66) | | 3.6  (1.6) | 2.4  (1.0) | |
| High pCO_2_ | Control | | 4 | 34.8 (0.1) | 2264  (1) | 2161  (7) | 7.69  (0.02) | 1002  (41) | | | 2036  (9) | | 92  (3) | | 2.2  (0.1) | 1.4  (0.1) | |
|  | *A. amadelpha* | | 2 | 35.0 (0.1) | 2267  (13) | 2134  (9) | 7.77  (0.01) | 799  (12) | | | 1999  (7) | | 109  (2) | | 2.6  (0.1) | 1.7  (0.03) | |
|  | *H. taenicola* | | 2 | 34.9 (0.1) | 1573  (444) | 1570  (329) | 7.29  (0.46) | 2314  (1573) | | | 1445  (336) | | 50  (43) | | 1.2  (1.0) | 0.8  (0.7) | |
