# Supplementary material for: Contrasting effects of ocean acidification on tropical fleshy and calcareous algae
Source: PeerJ. 2014 May 27;2:e411. doi: 10.7717/peerj.411 (PMC4045329; doi:10.7717/peerj.411)
Supplement: Table S4 — The mean (±SE) growth rate for each species across experiments standardized to the initial weight and the duration of the experiment. Sample size (n) is the number of replicates within a treatment per experiment. [file peerj-02-411-s004.docx]

**Table S4.** G**rowth rate of fleshy and calcareous algae.**

| Species | Year | Treatment | n | Growth  (mg g^-1^ d^-1^) |
| --- | --- | --- | --- | --- |
| **Fleshy macroalgae** |  |  |  |  |
| *Acanthophora spicifera* | 2010 | Ambient air | 6 | -35.23 ± 13.52 |
|  |  | High pCO_2_ | 6 | -12.21 ± 14.66 |
| *Avrainvillea amadelpha* | 2012 | Ambient air | 10 | 5.95 ± 2.49 |
|  |  | High pCO_2_ | 7 | -4.24 ± 2.76 |
| *Caulerpa serrulata* | 2010 | Ambient air | 6 | 1.98 ± 2.43 |
|  |  | High pCO_2_ | 6 | 19.41 ± 3.26 |
|  | 2011 | Ambient air | 4 | 7.51 ± 10.82 |
|  |  | High pCO_2_ | 3 | -16.43 ± 6.46 |
| *Dictyota bartayresiana* | 2011 | Ambient air | 5 | 11.09 ± 3.62 |
|  |  | High pCO_2_ | 5 | 22.60 ± 4.03 |
| *Hypnea pannosa* | 2011 | Ambient air | 3 | 1.31 ± 8.36 |
|  |  | High pCO_2_ | 4 | 18.2 ± 4.45 |
| **Upright calcareous algae** |  |  |  |  |
| *Dichotomaria marginata* | 2011 | Ambient air | 5 | 19.96 ± 3.66 |
|  |  | High pCO_2_ | 5 | 0.367 ± 3.57 |
| *Galaxaura rugosa* | 2010 | Ambient air | 6 | 1.38 ± 1.42 |
|  |  | High pCO_2_ | 6 | -9.08 ± 6.25 |
| *Halimeda opuntia* | 2009 | Ambient air | 4 | 6.59 ± 1.65 |
|  |  | High pCO_2_ | 4 | -7.96 ± 1.11 |
|  | 2011 | Ambient air | 5 | 25.37 ± 2.93 |
|  |  | High pCO_2_ | 5 | 12.40 ± 2.07 |
| *Halimeda taenicola* | 2009 | Ambient air | 4 | 8.02 ± 0.204 |
|  |  | High pCO_2_ | 4 | 0.885 ± 1.19 |
|  | 2010 | Ambient air | 6 | 43.32 ± 18.53 |
|  |  | High pCO_2_ | 6 | 37.52 ± 18.03 |
|  | 2012 | Ambient air | 10 | 23.31 ± 3.30 |
|  |  | High pCO_2_ | 10 | 25.97 ± 2.82 |
| **Crustose coralline algae** |  |  |  |  |
| *Lithophyllum* sp. | 2009 | Ambient air | 4 | 0.194 ± 0.033 |
|  |  | High pCO_2_ | 4 | -0.165 ± 0.081 |
|  | 2011 | Ambient air | 5 | 0.228 ± 0.076 |
|  |  | High pCO_2_ | 5 | -0.196 ± 0.097 |
| *Lithophyllum prototypum* | 2009 | Ambient air | 4 | 0.266 ± 0.135 |
|  |  | High pCO_2_ | 4 | -0.240 ± 0.122 |
