# Supplementary material for: Contrasting effects of ocean acidification on tropical fleshy and calcareous algae
Source: PeerJ. 2014 May 27;2:e411. doi: 10.7717/peerj.411 (PMC4045329; doi:10.7717/peerj.411)
Supplement: Table S5 — The mean (±SE) photosynthetic parameters across species in response to CO2 enrichment as estimated from rapid light curves with the equations of Platt, Gallegos & Harrison (1980). Sample size (n) is the number of replicate samples within a treatment, and repeats is the number of RLCs run for each sample. rETRMax, maximum relative electron transport rate (µM photon m−2 sec−1); α, photosynthetic efficiency or initial slope of the rapid light curve (µM electrons µM photons−1); β, photoinhibition µM electrons µM photons−1). [file peerj-02-411-s005.docx]

**Table S5.** **Photosynthetic parameters in response to treatment conditions**

| Species | Year | Treatment | n | Repeats | rETR_Max_ | α | β |
| --- | --- | --- | --- | --- | --- | --- | --- |
| **Fleshy Macroalgae** |  |  |  |  |  |  |  |
| *Acanthophora spicifera* | 2010 | Ambient | 6 | 2 | 12.76 ± 1.49 | 0.30 ± 0.04 | 0.010 ± 0.002 |
|  |  | High pCO_2_ | 5 | 2 | 8.33 ± 0.60 | 0.32 ± 0.01 | 0.009 ± 0.002 |
| *Caulerpa serrulata* | 2010 | Ambient | 6 | 2 | 17.30 ± 1.19 | 0.44 ± 0.004 | 0.07 ± 0.009 |
|  |  | High pCO_2_ | 6 | 2 | 18.02 ± 0.66 | 0.44 ± 0.02 | 0.06 ± 0.005 |
| *Caulerpa serrulata* | 2011 | Ambient | 5 | 1 | 3.76 ± 0.59 | 0.30 ± 0.05 | 0 |
|  |  | High pCO_2_ | 4 | 1 | 3.19 ± 1.92 | 0.23 ± 0.08 | 0.007 ± 0.007 |
| *Dictyota bartayresiana* | 2011 | Ambient | 5 | 1 | 23.43 ± 3.24 | 0.28 ± 0.03 | 0.0004 ± 0.0004 |
| . |  | High pCO_2_ | 5 | 1 | 32.86 ± 5.13 | 0.27 ± 0.03 | 0 |
| *Hypnea pannosa* | 2011 | Ambient | 5 | 1 | 35.01 ± 6.40 | 0.25 ± 0.02 | 0.01 ± 0.008 |
|  |  | High pCO_2_ | 4 | 1 | 29.00 ± 1.41 | 0.25 ± 0.04 | 0.02 ± 0.009 |
| **Upright calcareous algae** |  |  |  |  |  |  |  |
| *Dichotomara marginata* | 2011 | Ambient | 5 | 1 | 12.56 ± 3.33 | 0.26 ± 0.03 | 0.0009 ± 0.0007 |
|  |  | High pCO_2_ | 5 | 1 | 15.48 ± 1.34 | 0.25 ± 0.02 | 0.003 ± 0.002 |
| *Galaxaura rugosa* | 2010 | Ambient | 6 | 2 | 12.95 ± 0.56 | 0.23 ± 0.005 | 0.02 ± 0.005 |
|  |  | High pCO_2_ | 6 | 2 | 17.00 ± 0.82 | 0.25 ± 0.01 | 0.02 ± 0.003 |
| *Halimeda opuntia* | 2009 | Ambient | 4 | 3 | 15.17 ± 0.24 | 0.38 ± 0.008 | 0.04 ± 0.003 |
|  |  | High pCO_2_ | 4 | 3 | 12.72 ± 0.88 | 0.35 ± 0.01 | 0.04 ± 0.005 |
| *Halimeda opuntia* | 2011 | Ambient | 5 | 1 | 5.32 ± 1.00 | 0.47 ± 0.05 | 0.002 ± 0.002 |
|  |  | High pCO_2_ | 4 | 1 | 7.09 ± 1.66 | 0.38 ± 0.06 | 0.008 ± 0.006 |
| *Halimeda taenicola* | 2009 | Ambient | 4 | 3 | 10.71 ± 0.97 | 0.39 ± 0.02 | 0.03 ± 0.002 |
|  |  | High pCO_2_ | 4 | 3 | 10.99 ± 0.96 | 0.39 ± 0.02 | 0.03 ± 0.004 |
| *Halimeda taenicola* | 2010 | Ambient | 6 | 2 | 11.75 ± 1.60 | 0.47 ± 0.04 | 0.04 ± 0.010 |
|  |  | High pCO_2_ | 6 | 2 | 11.19 ± 0.77 | 0.37 ± 0.02 | 0.08 ± 0.010 |
| **Crustose coralline algae** |  |  |  |  |  |  |  |
| *Lithophyllum* sp. | 2009 | Ambient | 4 | 3 | 4.02 ± 0.43 | 0.26 ± 0.01 | 0 |
|  |  | High pCO_2_ | 4 | 3 | 7.22 ± 1.46 | 0.26 ± 0.03 | 0 |
| *Lithophyllum* sp. | 2011 | Ambient | 5 | 1 | 15.15 ± 1.32 | 0.17 ± 0.03 | 0.001 ± 0.001 |
|  |  | High pCO_2_ | 5 | 1 | 15.85 ± 3.09 | 0.15 ± 0.01 | 0 |
| *Lithophyllum prototypum* | 2009 | Ambient | 4 | 3 | 3.40 ± 0.34 | 0.34 ± 0.03 | 0 |
|  |  | High pCO_2_ | 4 | 3 | 3.66 ± 0.64 | 0.36 ± 0.02 | 0 |
