# Supplementary material for: Contrasting effects of ocean acidification on tropical fleshy and calcareous algae
Source: PeerJ. 2014 May 27;2:e411. doi: 10.7717/peerj.411 (PMC4045329; doi:10.7717/peerj.411)
Supplement: Supplemental Information 1 — Details of the statistical analysis involved with the meta-analysis. [file peerj-02-411-s006.docx]

**SUPPLEMENTAL INFORMATION**

**METHODS**

Meta-analysis techniques were used to explore the consistency of algal functional group response to CO_2_ enrichment manipulations (Cooper et al., 1994). Because different species were examined in different years, a random effects model was used to quantify the across experiment variation and combine species-specific estimates of effect size (in the original, shared metric of proportional growth) for each functional group of algae (fleshy macroalgae, upright calcareous algae, and crustose coralline algae (CCA)). The one-sided hypothesis that the common mean effect size *θ* for growth or calcification*,* with variance *ν_i_*^*^, was greater than zero for fleshy macroalgae and less than zero for either functional form (calcified macroalgae or CCA) of calcified algae was tested based on an *a priori* expectation that CO_2_ enrichment will fertilize productivity but inhibit calcification. For photo-physiological response variables, a two-sided test was used as there was no expectation for the direction of response. Experiments for certain species that were tested more than once were combined across years and experiments where algae reproduced were excluded from this analysis.

The estimate of *θ_i_* (defined as the mean difference between growth, calcification, or photophysiological parameters in experimental and control treatments, $\bar{T.}$) had a different sample size *k* reflecting the total number of species represented in each functional group. The total variability of the mean effect size, *ν_i_*^*^ reflects the sum of both conditional variation within (variance of *T_i_*, defined as *ν_i_*) and across experiments (*σ_θ_*^2^).

For each species, the mean difference between treatment (CO_2_ enrichment) and control (ambient air) group means was calculated as difference in means between groups, or $T_{i} = \bar{Y}_{i}^{T}-\bar{Y}_{i}^{C}$. Because the standard deviations of each group were known, the pooled within-group variance was used for each experimental *T_i_*:

$$s_{pi}^{2}=\left[ \left( n_{i}^{T}-1 \right)\left( s_{i}^{T} \right)^{2}+\left( n_{i}^{C}-1 \right)\left( s_{i}^{C} \right)^{2} \right]/\left[ n_{i}^{T}+n_{i}^{C}-2 \right]$$

where (*s_i_^T^*)^2^ is the variance (and *n_i_^T^* is the sample size) of the treatment group in the *i*th experiment, and (*s_i_^C^*)^2^ is the variance (and *n_i_^C^* is the sample size) of the control group. Thus, the conditional variation within experiments was estimated as *ν_i_* = *σ_pi_*^2^(1/*n_i_^T^* + 1/*n_i_^C^*) with *σ_pi_*^2^.

For each functional group, the across experiments variance component was estimated as:

$$\hat{\sigma}_{\theta}^{2}=\left[ Q-\left( k-1 \right) \right]/c$$

where *Q* is the homogeneity test statistic to test the hypothesis that *σ_θ_*^2^ = 0 and *c* is a component of the expected value of *Q*. If the across treatment variance is homogenous, this estimate of the variance component will be exceedingly small and will not contribute to *ν_i_*^*^; only the conditional variation of *T_i_* is used in hypothesis testing.

The formal test statistic for our null hypotheses was $Z=\bar{T.}/{\left( \nu^{*}. \right)^{1/2}}$ with confidence limits $\theta_{L}=\bar{T.}-C_{\alpha/2}\left( \nu. \right)^{1/2}$ for fleshy macroalgae and $\theta_{U}=\bar{T.}+C_{\alpha/2}\left( \nu. \right)^{1/2}$ for calcareous algae. This random effects approach provides more conservative estimates of average effect size over independent experiments in the presence of unexplained heterogeneity arising from conducting studies in different years and under slightly different environmental conditions.

**SUPPLEMENTAL REFERENCES**

Cooper H, Hedges LV, Valentine JC. 1994. *The Handbook of Research Synthesis and Meta-*

*analysis*. Russell Sage Foundation.

Platt T, Gallegos CL, Harrison WG. 1980. Photoinhibition of photosynthesis in natural assemblages of marine phytoplankton. *Journal of Marine Research* **38**:687-701.
